# Croup

**Published:** 1890-03-01

**Authors:** 


### CROUP.

In the *New York Medical Journal*, Dr. A. Brothers, physician to the children's department of the Bellevue Hospital, gives some observations on the treatment of croup. He regards croup as a form of laryngeal stenosis tending to produce death by suffocation. It may be due (1) to a simple catarrhal laryngitis; (2) it may originate in a croupous bronchitis; (3) it may begin as a diphtheritic laryngitis. The first class of cases begin suddenly, usually in the night, and do well under a mild emetic, steam, bichloride of mercury, chlorate of potassium, and warm applications to the throat. In the second class of cases acute catarrhal bronchitis is simulated, but the child becoming worse, expectoration of membrane clears the diagnosis. An occasional vomit can do no harm, and may loosen the membrane. Bichloride of mercury and free stimulation are indicated. When stenosis becomes threatening, tracheotomy offers the better chance. Diphtheritic laryngitis may follow nasal diphtheria, and in such cases the author advises resort at once to the bichloride of mercury.

